# A bioinformatic analysis: the overexpression and clinical significance of FCGBP in ovarian cancer

**DOI:** 10.18632/aging.202601

**Published:** 2021-03-03

**Authors:** Kai Wang, Chenan Guan, Xianwen Shang, Xiang Ying, Shuangshuang Mei, Hanxiao Zhu, Liang Xia, Zeying Chai

**Affiliations:** 1 Taizhou Hospital of Zhejiang Province Affiliated to Wenzhou Medical University, Linhai 317000, Zhejiang Province, People’s Republic of China; 2 Department of Obstetrics and Gynecology, Taizhou Hospital of Zhejiang Province Affiliated to Wenzhou Medical University, Linhai 317000, Zhejiang Province, People’s Republic of China; 3 Department of Kidney Internal Medicine, Taizhou Hospital of Zhejiang Province Affiliated to Wenzhou Medical University, Linhai 317000, Zhejiang Province, People’s Republic of China; 4 Department of Neurosurgery, Zhejiang Cancer Hospital, Hangzhou 310022, People’s Republic of China

**Keywords:** Fc fragment of IgG binding protein, ovarian cancer, biomarker

## Abstract

Fc fragment of IgG-binding protein (FCGBP) is differentially expressed in various tumors. However, the correlation between FCGBP and immune cell infiltration in ovarian cancer remains unclear. FCGBP expression was analyzed using The Cancer Genome Atlas (TCGA) pan-cancer data, and the ovarian cancer expression profile was analyzed using the Gene Expression Omnibus database. The clinical prognostic value of FCGBP was evaluated using clinical survival data from TCGA. Enrichment analysis of FCGBP was performed using the R package clusterProfiler. Based on known immune cell infiltration scores for samples found in TCGA, we analyzed the association between immune cell infiltration level and FCGBP expression. FCGBP was highly expressed and associated with poorer overall survival (p = 0.00051) and disease-specific survival (p = 0.0012) in ovarian cancer and other tumors. Additionally, high FCGBP expression correlated significantly with immune-related gene sets, including those involved in chemokine signaling pathways and innate and adaptive immunity. Further analysis showed that M2 macrophage infiltration increased and M1 macrophage infiltration decreased in tissues with high FCGBP expression. Our study suggests that FCGBP contributes to M2 macrophage polarization by acting as an oncogene in ovarian cancer. FCGBP may represent a clinically helpful biomarker for predicting overall survival of ovarian cancer patients.

## INTRODUCTION

Ovarian cancer represents one of the major gynecologic malignant tumors. It is prone to relapse and metastasis as well as drug resistance development and exhibits a high mortality rate [1]. Currently, there is a lack of effective early tumor markers and diagnostic methods for ovarian cancer. With the rapid development of high-throughput sequencing technology and transcriptomic research, an increasing number of key driver genes have been discovered. However, there remains a clear need to identify additional key driver genes, particularly those that could affect the composition of the immune microenvironment in ovarian cancer.

Fc fragment of IgG-binding protein (FCGBP) may represent one such protein marker as its expression is low in some tumors, while high in others [2–4]. For example, expression of FCGBP is low in gallbladder cancer, in which it serves as a key regulator of tumor growth factor 1 (TGF-1)-induced epithelial-mesenchymal transition [5]. Meanwhile, its low expression in prostate cancer is reportedly correlated with disease progression [6]. In contrast, FCGBP is highly expressed in colorectal cancer, particularly in metastatic tissues. Moreover, increased expression of FCGBP significantly decreases the overall survival (OS) of colorectal cancer patients [7]. However, the role of FCGBP in ovarian cancer remains unknown.

In our study, we evaluated the expression of FCGBP in various tumors described in three cohorts including The Cancer Genome Atlas (TCGA), Genotype-Tissue Expression (GTEx), and Gene Expression Omnibus (GEO), as well as its correlation with patient prognosis. We found that FCGBP was over-expressed in ovarian cancer. High FCGBP expression was correlated with poorer OS and disease-specific survival (DSS) of ovarian cancer patients. Furthermore, FCGBP was predicted to be involved in chemokine signaling pathways, as well as the innate and adaptive immune systems. Moreover, considering that the infiltration of immune cells (especially macrophages) is important for the overall survival of patients with ovarian cancer [8, 9], we examined the association between FCGBP expression and the immune cell infiltration level and found that M2 macrophage infiltration increased, while M1 macrophage infiltration decreased in tissues with high FCGBP expression. Our results suggest potential functional role of FCGBP in ovarian cancer, thereby highlighting a mechanistic basis whereby FCGBP influences M2 macrophage polarization in the tumor microenvironment.

## RESULTS

### Pan-cancer FCGBP expression analysis

**Figure 1:**
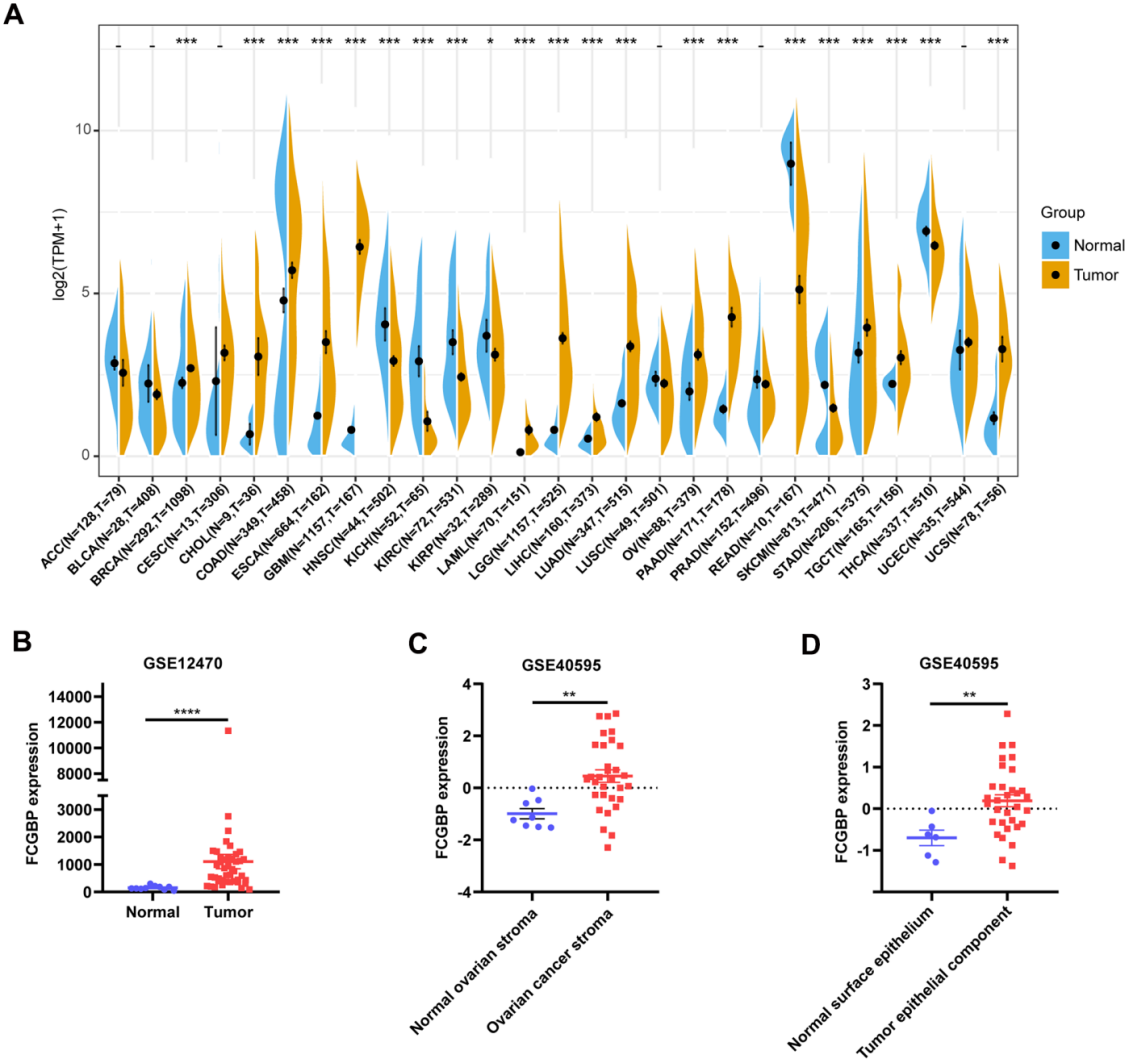
**Pan-cancer FCGBP expression analysis.** (**A**) FCGBP expression in tumor and normal tissues in TCGA and GTEx pan-cancer data. (**B**) FCGBP expression in tumor and normal tissues in ovarian cancer from GSE12470. (**C**) FCGBP expression in normal ovarian stroma and ovarian cancer stroma from GSE40595. (**D**) FCGBP expression in normal ovarian surface epithelium and ovarian cancer epithelial component from GSE40595.

**Figure 2:**
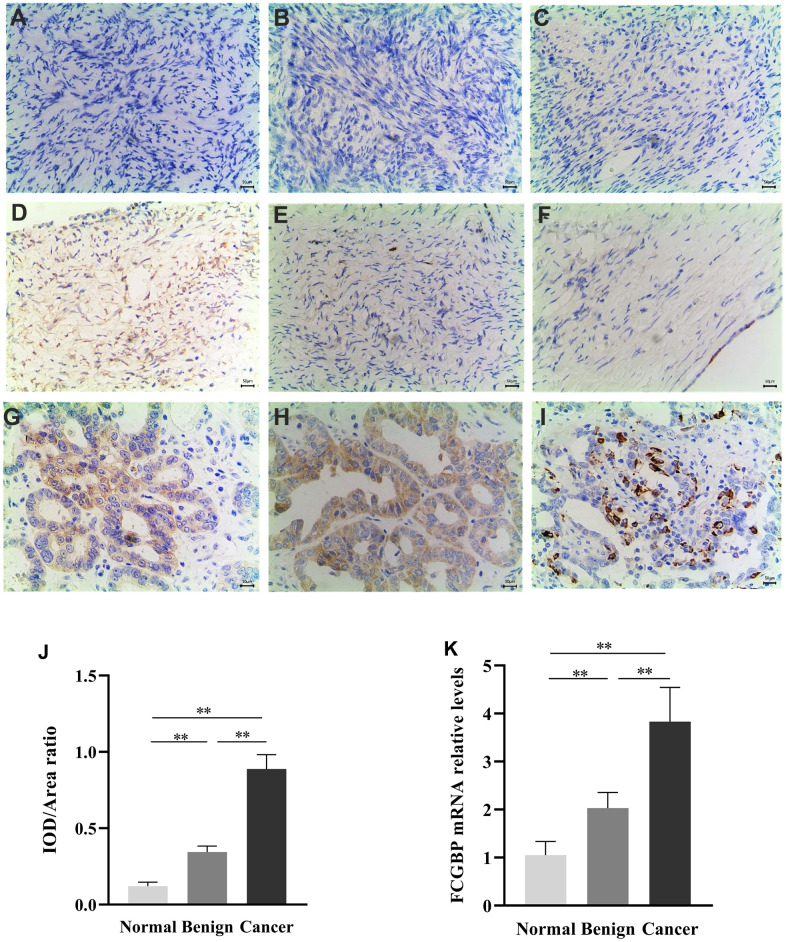
**Expression of FCGBP in ovarian cancer tissues.** (**A**–**C**) Representative images of immunohistochemistry showing FCGBP expression in normal ovarian tissues. (**D**–**F**) Representative images of immunohistochemistry showing FCGBP expression in benign ovarian cancer tissues. (**G**–**I**) Representative immunohistochemistry images showing FCGBP expression in ovarian cancer tissues. (**J**) IOD/area ratio of the indicated immunohistochemistry images. (**K**) qRT-PCR analysis of FCGBP expression in the indicated groups.

We first assessed FCGBP expression in pan-cancer data from TCGA and GTEx. The analysis revealed FCGBP expression to be higher in 14 tumors, including BRCA, CHOL, COAD, ESCA, GBM, AML, LGG, LIHC, LUAD, OV, PAAD, STAD, TGCT, and UCS. In contrast, its expression was low in HNSC, KICH, KIRC, KIRP, READ, SKCM, and THCA (Figure 1A). In addition, FCGBP was highly expressed in ovarian cancer in GSE12470 and GSE40595 (Figure 1B−1D). We further confirmed the expression of FCGBP in ovarian cancer via immunohistochemistry (Figure 2A−2J) and qRT-PCR (Figure 2K). The results confirmed that FCGBP was overexpressed in ovarian cancer tissues compared with normal ovarian tissues.

### Association between FCGBP expression and cancer patient prognosis

**Figure 3:**
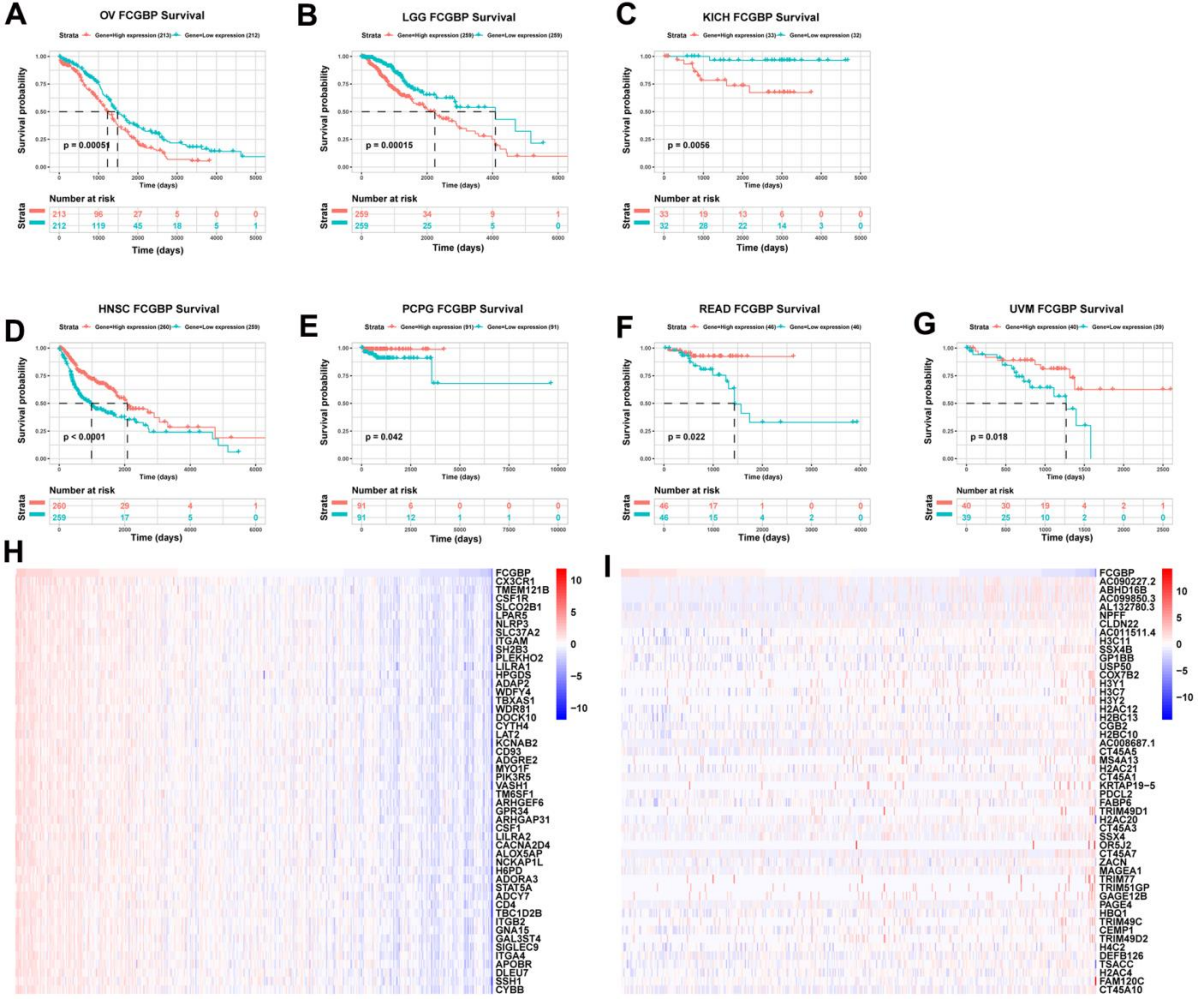
**Association between FCGBP expression and cancer prognosis.** (**A**–**G**) Kaplan–Meier analysis of overall survival in TCGA. Results with logrank p <0.05 are shown. (**H**) Top 50 genes most positively associated with FCGBP are shown in a heatmap. (**I**) Top 50 genes most negatively associated with FCGBP are shown in a heatmap.

To evaluate the value of FCGBP in predicting the prognosis of cancer patients, the association between FCGBP expression and OS, DSS, and the progression-free interval was analyzed in TCGA cohort. For OS, higher expression of FCGBP was significantly associated with reduced OS in OV (p = 0.00051), LGG (p = 0.00015), and KICH (p = 0.0056) (Figure 3A−3C). In contrast, HNSC (p < 0.0001), PCPG (p = 0.042), READ (p = 0.022), and UVM (p = 0.018) displayed trends toward improved survival with increasing FCGBP expression (Figure 3D−3G). Higher FCGBP expression was significantly associated with increased DSS in HNSC (p < 0.0001), BRCA (p = 0.03), PCPG (p = 0.019), and KIRC (p = 0.046; Supplementary Figure 1A−1D) and with poor DSS in OV (p = 0.0012), KICH (p = 0.02), and LGG (p = 0.00012; Supplementary Figure 1E−1G). In addition, the progression-free interval increased in the high FCGBP expression group in THCA (p = 0.0042), UCEC (p = 0.039), UVM (p = 0.012), and HNSC (p < 0.0001; Supplementary Figure 1H−1K), while it was reduced in KICH (p = 0.041) and LGG (p < 0.0001; Supplementary Figure 1L−1M).

### Correlation and enrichment analyses

**Figure 4:**
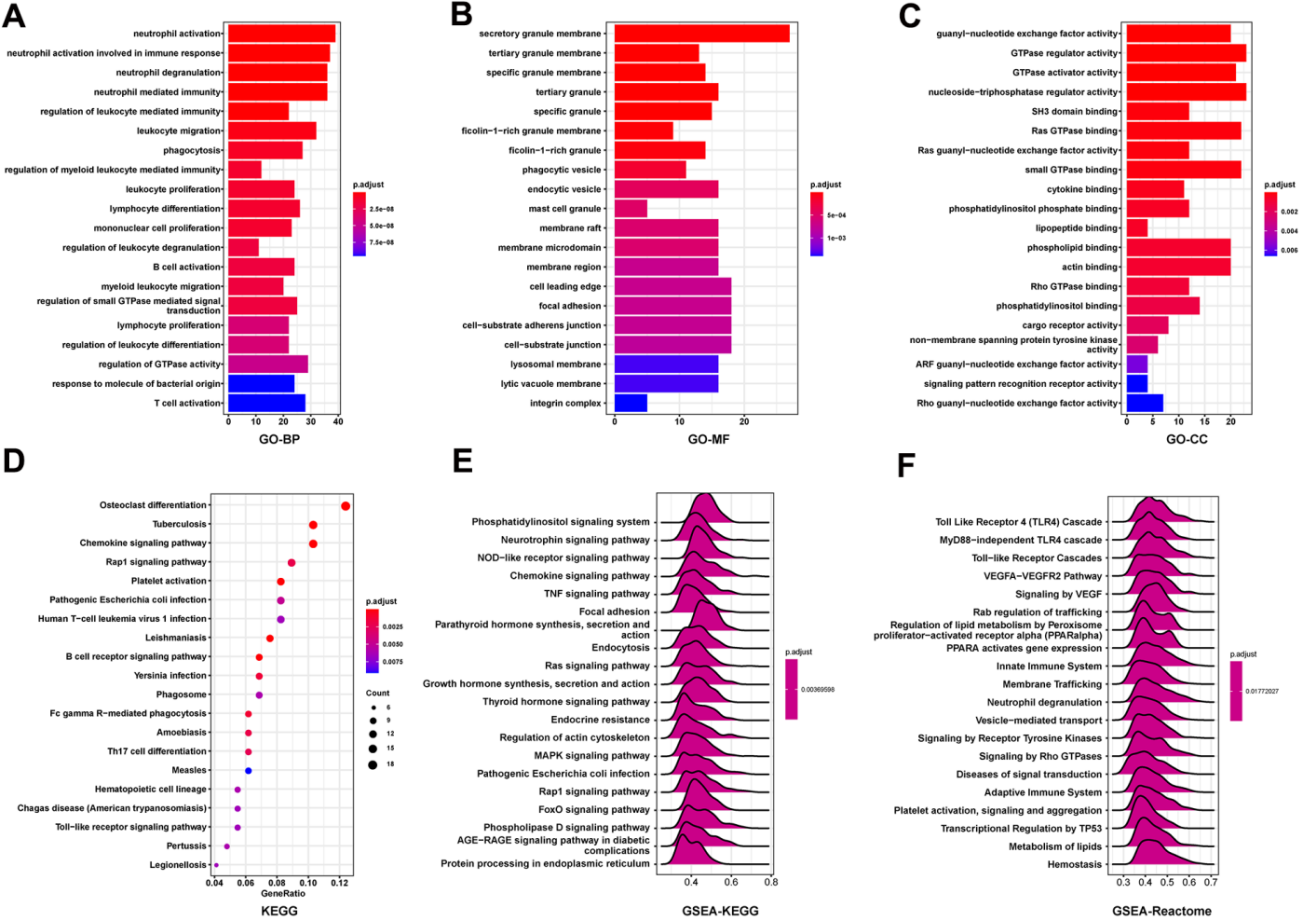
**Function and pathway enrichment analyses of FCGBP in ovarian cancer.** (**A**–**C**) Significant Gene Ontology terms of the top 300 genes most positively associated with FCGBP, including biological processes (BP), molecular function (MF), and cell component (CC). (**D**) Significant KEGG pathways of the top 300 genes most positively associated with FCGBP. (**E**, **F**) Significant GSEA results of the top 300 genes most positively associated with FCGBP, including KEGG pathways (**E**) and Reactome pathways (**F**).

To predict the function of FCGBP, including associated pathways, we performed a correlation analysis between FCGBP and other genes in ovarian cancer using TCGA data (Figure 3H−3I). The top 300 genes that associated most positively with FCGBP were selected for enrichment analysis. We further explored the potential functional pathways based on the top 300 genes using the clusterProfiler R package. Functional enrichment and Gene Ontology (GO) analysis revealed that FCGBP was primarily associated with immune-related gene terms, including regulation of leukocyte-mediated immunity, leukocyte migration, leukocyte proliferation, B cell activation, and T cell activation (Figure 4A−4C). In addition, Kyoto Encyclopedia of Genes and Genomes (KEGG) pathway analysis indicated an enrichment and crosstalk of the top 300 genes in chemokine, RAP1, and B cell receptor signaling pathways, as well as human T cell leukemia virus 1 infection (Figure 4D). Gene Set Enrichment Analysis (GSEA) was used to search for KEGG and Reactome pathways, which revealed that the phosphatidylinositol, neurotrophin, NOD-like receptor, and chemokine signaling pathways were significantly enriched (Figure 4E). In addition, Toll-like receptor 4 (TLR4) cascade, MyD88-independent TLR4 cascade, Toll-like receptor cascades, and VEGFA−VEGFR2 pathway were significantly enriched by Reactome pathway analysis (Figure 4F). These results suggest that FCGBP is associated with many malignancy-related pathways in ovarian cancer, especially immune-related pathways.

### Correlation between immune cell infiltration and FCGBP

**Figure 5:**
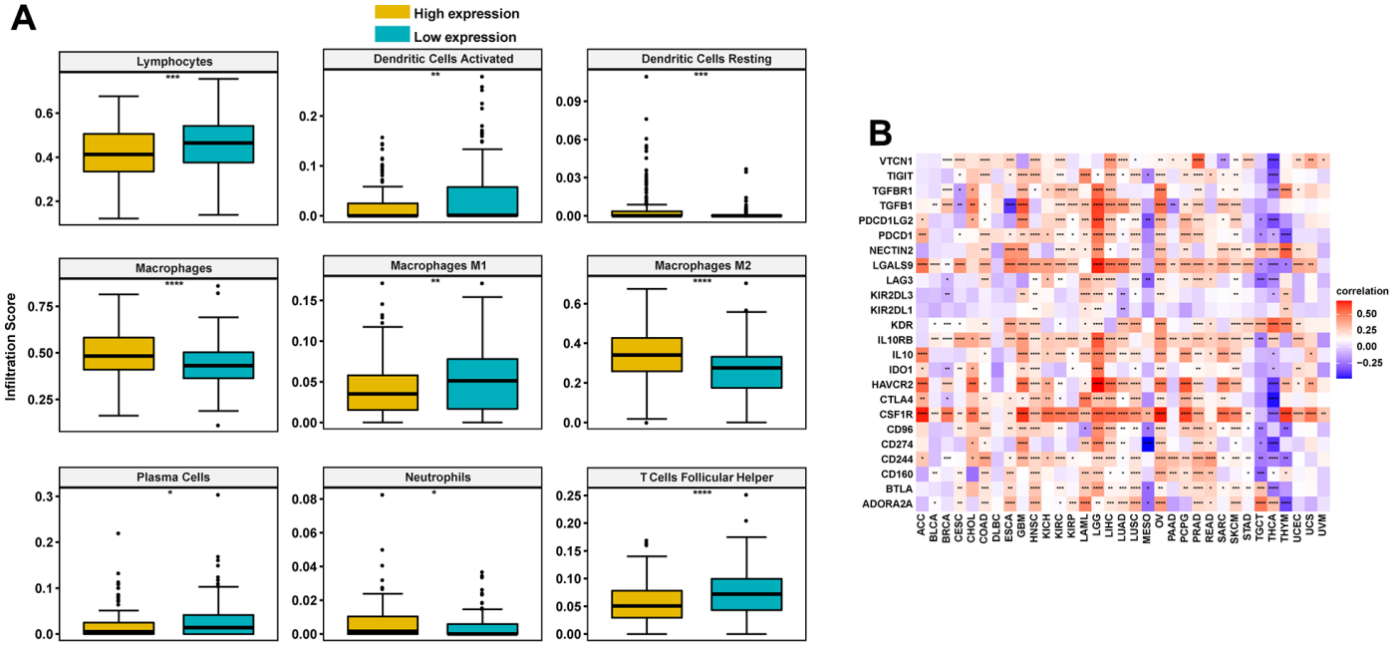
**Correlation between immune cell infiltration and FCGBP in ovarian cancer.** (**A**) Immune cell infiltration level in the high FCGBP expression group and low FCGBP expression group in TCGA cohort. (**B**) Correlations between FCGBP and immunosuppressive genes are shown in a heatmap, where red represents positive correlation, blue represents negative correlation; the deeper the color, the stronger the correlation.

We further assessed the immune cell infiltration score of TCGA ovarian cancer and found that the M2 macrophage infiltration level was high, while that of M1 macrophages was low in the high-FCGBP expression group (Figure 5A). This indicates that high expression of FCGBP promotes the polarization of macrophages, which is closely related to the immunosuppressive state of the tumor [10]. We further analyzed the relationship between FCGBP and immunosuppressive genes using TCGA pan-cancer data. FCGBP was highly positively correlated with immunosuppressive genes in most tumors, including ovarian cancer (Figure 5B). These results suggest that high expression of FCGBP is closely related to the immunosuppressive status of ovarian cancer.

### FCGBP expression was associated with macrophage infiltration and polarization

**Figure 6:**
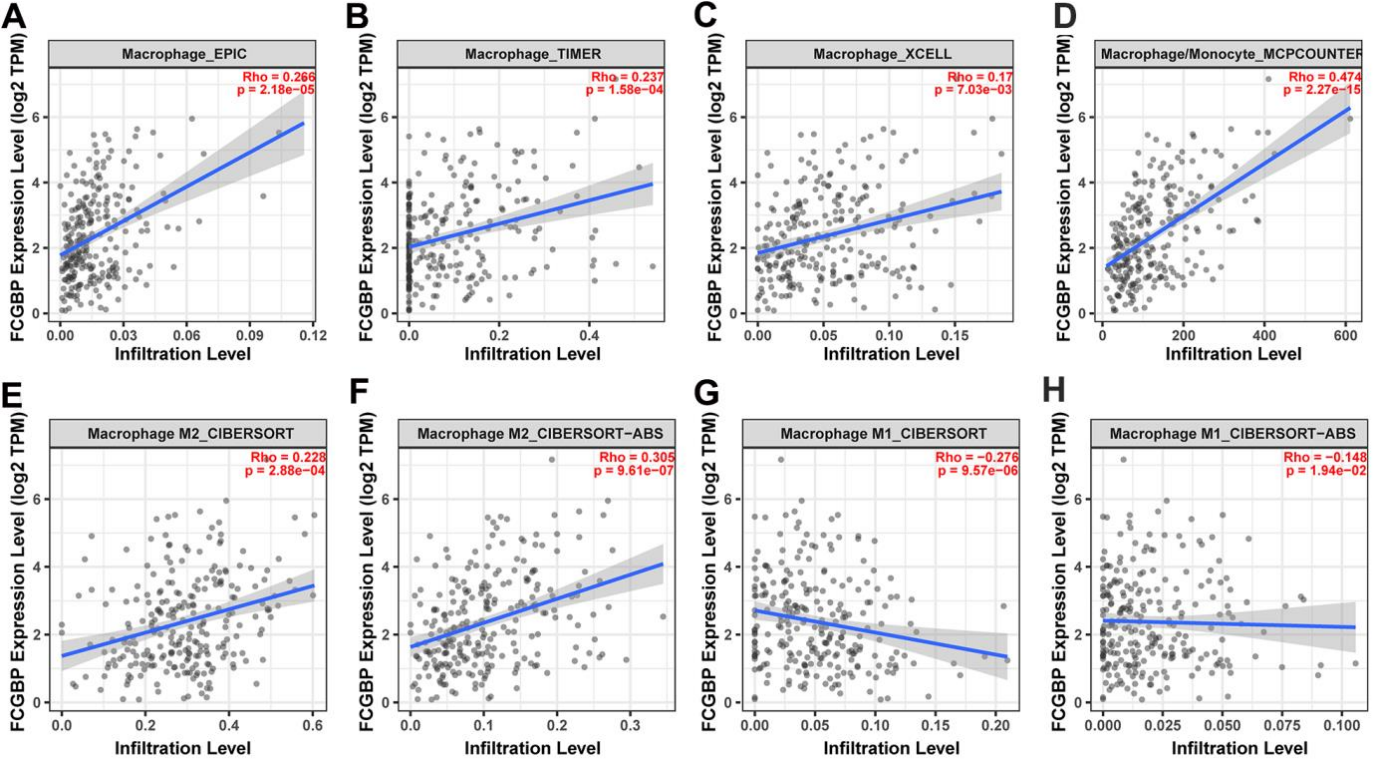
**Correlation between FCGBP expression and macrophage infiltration and polarization.** (**A**–**D**) Correlation between FCGBP expression and macrophage infiltration levels using four different algorithms: EPIC, TIMER, XCELL, and MCPCOUNTER. (**E**, **F**) Correlation between FCGBP expression and M2-like macrophage infiltration levels using two different algorithms: CIBERSOFT and CIBERSOFT-ABS. (**G**, **H**) Correlation between FCGBP expression and M1-like macrophage infiltration levels using two different algorithms: CIBERSOFT and CIBERSOFT-ABS.

**Figure 7:**
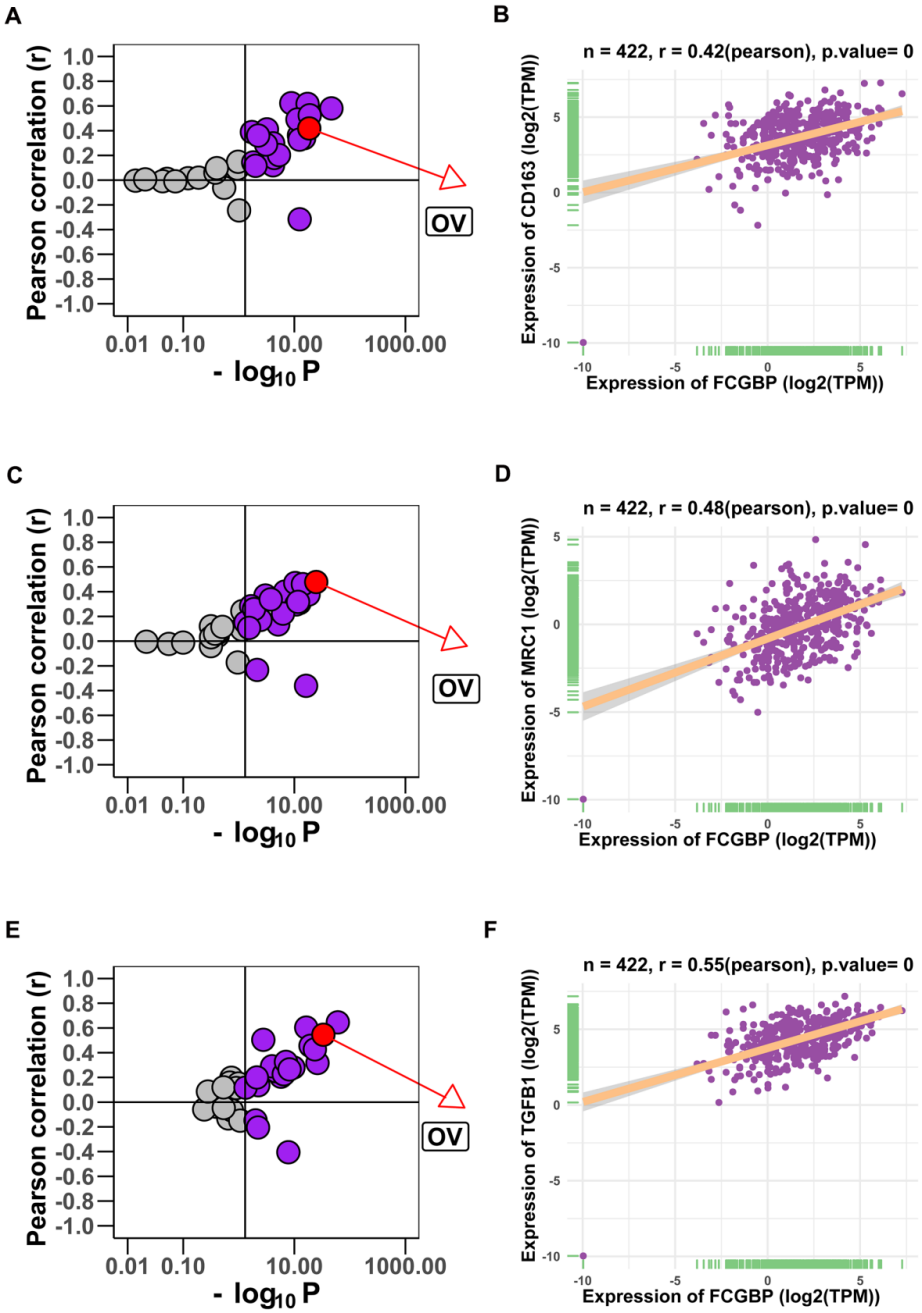
**Correlation between FCGBP expression and gene markers of M2-like macrophages.** (**A**, **B**) Correlation between FCGBP expression and CD163 in pan-cancer (**A**) and ovarian cancer (**B**). Each circle represents a type of cancer; purple circles represent meaningful correlations (Pearson p < 0.05). (**C**, **D**) Correlation between FCGBP expression and MRC1 in pan-cancer (**C**) and ovarian cancer (**D**). (**E**, **F**) Correlation between FCGBP expression and TGFB1 in pan-cancer (**E**) and ovarian cancer (**F**).

We further validated our results using the TIMER2 (http://timer.cistrome.org/) database and found that FCGBP expression was positively correlated with the macrophage infiltration level using four different algorithms (Figure 6A−6D). For macrophage polarization, the expression of FCGBP was positively correlated with M2 polarization (Figure 6E−6F) and negatively correlated with M1 polarization (Figure 6G−6H). Moreover, analysis using TCGA pan-cancer data provided the same results: high expression of FCGBP correlated well with macrophage infiltration and polarization in ACC (Supplementary Figure 2A−2C), BRCA (Supplementary Figure 2D−2F), MESO (Supplementary Figure 2G−2I), PAPG (Supplementary Figure 2J−2L), and SARC (Supplementary Figure 2M−2O). In addition, FCGBP expression correlated significantly with the markers of M2-like macrophages in pan-cancer and ovarian cancer, including CD163 (Figure 7A−7B), MRC1 (Figure 7C−7D), and TGFB1 (Figure 7E−7F). These results indicate that high FCGBP expression is associated with macrophage infiltration and polarization in addition to the immunosuppressive microenvironment in ovarian cancer.

## DISCUSSION

FCGBP is involved in the progression of many diseases; however, it has not been extensively studied in various tumors. Therefore, it is urgent to clarify the role of FCGBP in cancer prognosis, progression, and treatment. Previous studies have reported contradictory roles for FCGBP in different tumors. For instance, it is upregulated in HNSC patients with HPV infection, in which its upregulation correlates with longer survival time [11], which is consistent with our results. Meanwhile, in osteosarcoma and colon cancer, FCGBP is downregulated in tumor tissues compared with normal tissues, as well as in metastatic tissues compared with non-metastatic tissues [12, 13].

Based on our results, the FCGBP expression levels and prognostic function in pan-cancer, using TCGA and GTEx data from UCSC Xena, showed that FCGBP, compared to normal tissues, was highly expressed in BRCA, CHOL, COAD, ESCA, GBM, LAML, LGG, LIHC, LUAD, OV, PAAD, STAD, TGCT, and UCS, whereas low expression was observed in HNSC, KICH, KIRC, KIRP, READ, SKCM, and THCA. In addition, FCGBP was highly expressed in ovarian cancer in the GSE12470 and GSE40595 datasets. The difference in FCGBP expression levels in different tumor types may reflect distinct underlying functions and mechanisms. We further found that overexpression of FCGBP generally predicted poor prognosis for patients with tumors with high FCGBP expression, such as OV and LGG. In contrast, its low expression was correlated with poor prognosis in HNSC and READ. These results suggest that FCGBP is a potential biomarker for predicting the prognosis of tumor patients.

Tumor microenvironment immune cells constitute a key factor of tumor tissues with increasing evidence supporting their clinicopathological significance in predicting survival status and therapeutic efficacy of tumor patients [14–17]. Specifically, the infiltration level of TAM accelerates the progression of ovarian cancer [18, 19]. TAM consist primarily of M2 macrophages, likely due to exposure to complex factors in the tumor microenvironment [20, 21]. GO results showed that FCGBP was closely associated with immune-related pathways, including regulation of leukocyte-mediated immunity, leukocyte migration, leukocyte proliferation, B cell activation, and T cell activation. Meanwhile, KEGG analysis indicated that FCGBP was involved in the chemokine signaling pathway, RAP1 signaling pathway, human T cell leukemia virus 1 infection, and B cell receptor signaling pathway in ovarian cancer. By analyzing the relationship between FCGBP and immune cell infiltration, we found that the M2 macrophage infiltration level was significantly higher in the high FCGBP expression group; in this group, the M1 macrophage infiltration level was lower in ovarian cancer. Moreover, the correlation between FCGBP and immunosuppressive gene expression indicates that FCGBP plays a key role in regulating tumor immunology.

In summary, FCGBP likely plays an important role in immune cell infiltration and may represent a valuable prognostic biomarker for ovarian cancer.

## MATERIALS AND METHODS

### Data collection and analysis

FCGBP expression and clinical data of TCGA pan-cancer data and GTEx were obtained from the UCSC Xena database (https://xenabrowser.net/datapages/). The full names of TCGA tumor abbreviations were supplied in Supplementary Table 1. To evaluate FCGBP expression, tumor tissues were obtained from TCGA, and normal tissues were combined with normal tissues from the TCGA and GTEx databases. Ovarian cancer microarray data were obtained from the GEO database, including GSE12470 (Platform: GPL887) and GSE40595 (Platform: GPL570).

### Correlation and enrichment analyses

Correlation analysis between FCGBP and other mRNAs in ovarian cancer was performed using TCGA data, and the Pearson correlation coefficient was calculated. The top 300 genes most positively associated with FCGBP were selected for enrichment analysis to reflect the role of FCGBP. Gene Ontology (GO) analysis was performed using EnrichGO function in the R package “clusterProfiler”. Kyoto Encyclopedia of Genes and Genomes (KEGG) analysis was performed using the EnrichKEGG function of the R package “clusterProfiler”. Gene Set Enrichment Analysis (GSEA) was performed using the gseGO, gseKEGG, and gsePathway functions of the R package “clusterProfiler”.

### Immune cell infiltration

We downloaded the immune cell infiltration scores of TCGA pan-cancer obtained previously [22] that had been estimated using CIBERSORT [23]. To compare the level of immune cell infiltration, Samples from TCGA were divided into two groups (high FCGBP and low FCGBP) based on the median FCGBP expression level.

### qRT-PCR

TRIzol® Plus RNA Purification Kit (Invitrogen, Carlsbad, CA, USA) was used to perform total RNA extraction. First-strand cDNA was synthesized using SuperScript™ III First-Strand Synthesis SuperMix for qRT-PCR (Invitrogen, Carlsbad, CA, USA). Then, Power SYBR® Green PCR Master Mix kit (Applied Biosystems, USA) was used to perform real-time PCR. The primers used were as follows: human *GAPDH*-Forward: 5ʹ-CCATGACAACTTTGGTATCGTGGAA-3ʹ; human *GAPDH*-Reverse: 5ʹ-GGCCATCACGCCACAGTTTC-3ʹ; human *FCGBP*-Forward: 5ʹ-GCAGTGAGTTCTCGTATGCTGAA-3ʹ; human *FCGBP*- Reverse: 5ʹ-GAAGGTGAGCAGTCCCAAGTT-3ʹ.

### Immunohistochemistry

Human ovarian tumor specimens were obtained from the Department of Obstetrics and Gynecology, Taizhou Hospital of Zhejiang Province, Wenzhou Medical University. All experiments involving human tissues were in accordance with the principles of the Declaration of Helsinki and were approved by the Institutional Review Board of Taizhou Hospital. The primary antibodies and antigen retrieval regimes used were as follows: anti-FCGBP (Abcam [Ab121202], Cambridge, UK). The IOD/area ratio was calculated using ImagePro Plus 6.0. Statistical analyses were conducted using the GraphPad Prism 8.0.1 software. Statistical significance was evaluated using two-tailed t-tests: *p < 0.05, **p < 0.01, and ***p < 0.001.

### Ethics approval and consent to participate

Informed consent was obtained from all individual participants for whom identifying information was included in this article.

### Consent for publication

All the authors report no disclosures relevant to the manuscript.

### Data availability statements

The datasets generated and/or analyzed during the current study are available from the corresponding author upon reasonable request.

## Supplementary Material

Supplementary Figures

Supplementary Table 1
