# Supplementary material for: A bioinformatic analysis: the overexpression and clinical significance of FCGBP in ovarian cancer
Source: Aging (Albany NY). 2021 Mar 3;13(5):7416–29. doi: 10.18632/aging.202601 (PMC7993703; doi:10.18632/aging.202601)
Supplement: Supplementary Figures [file aging-13-202601-s001.pdf]

SUPPLEMENTARY FIGURES

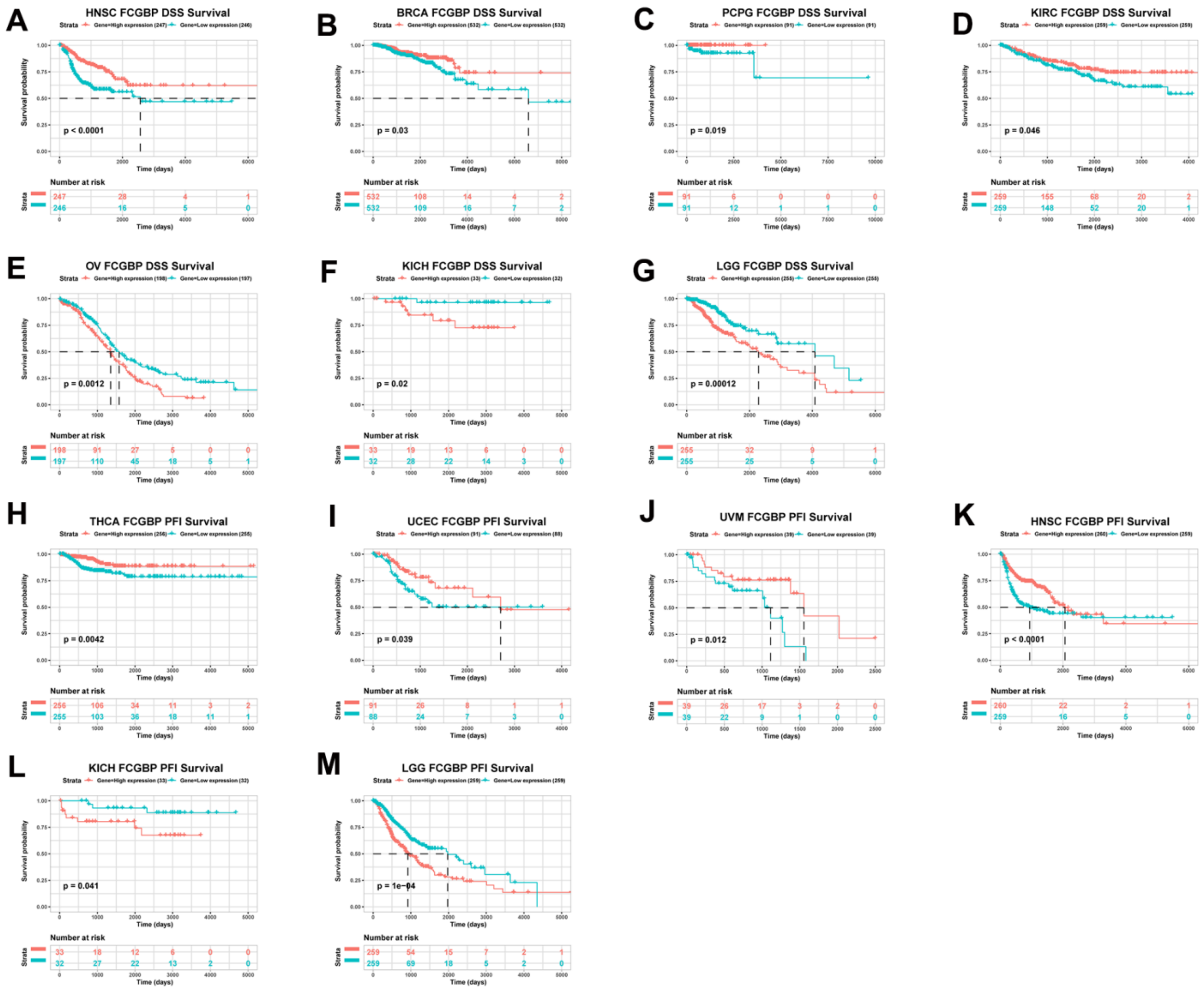

Supplementary Figure 1. The association between FCGBP expression and cancer patient prognosis. (**A**–**G**), Kaplan–Meier analysis of disease-specific survival in TCGA, Meaningless results were not shown. (**H**–**M**), Kaplan–Meier analysis of progression free interval in TCGA, Meaningless results were not shown.

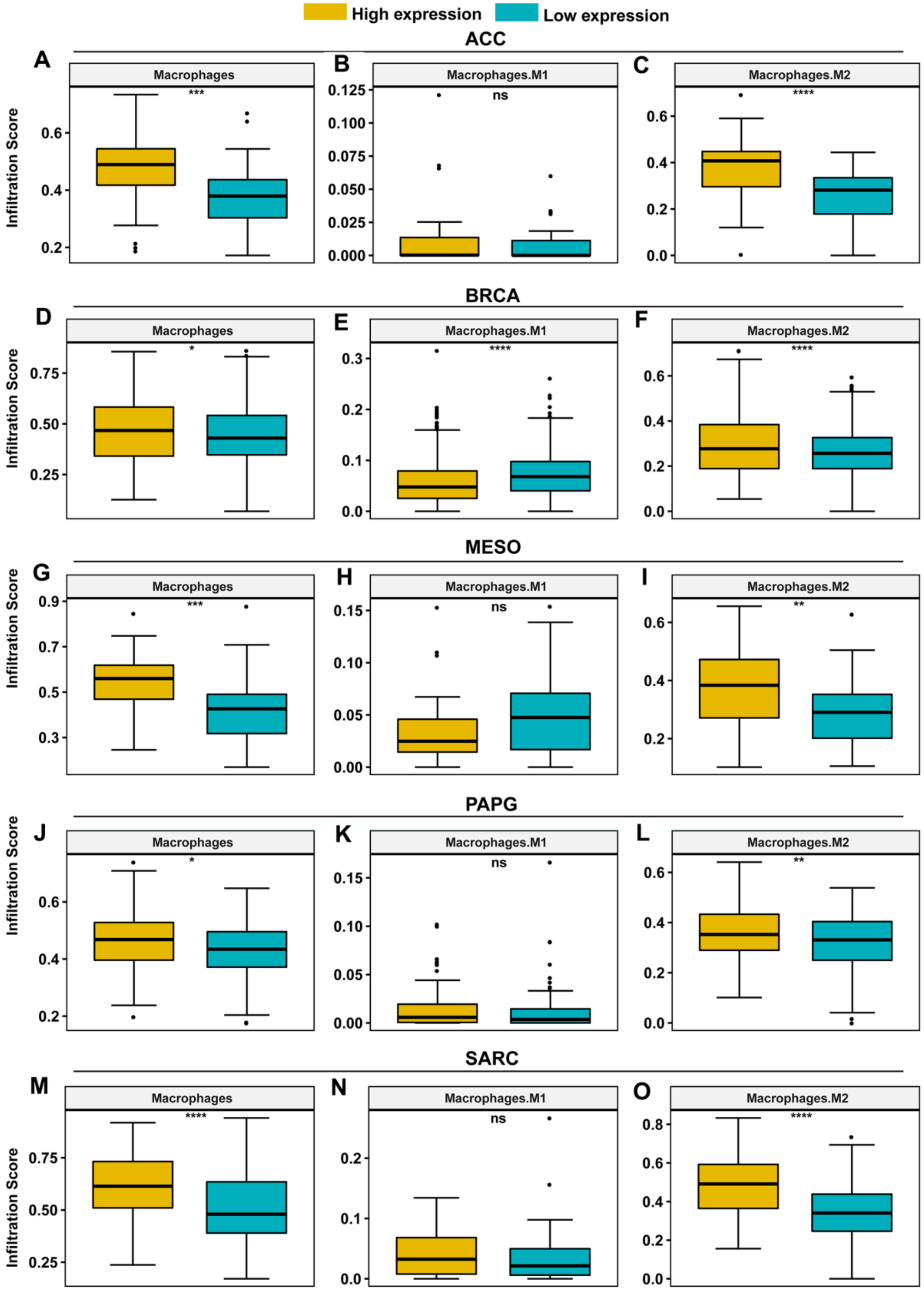

Supplementary Figure 2. Correlation between immune cell infiltration and FCGBP in pan-cancer. (**A**–**C**) Immune cell infiltration level in the high and low FCGBP expression groups in the ACC cohort. (**D**–**F**) Immune cell infiltration level in the high and low FCGBP expression groups in the BRCA cohort. (**G**–**I**) Immune cell infiltration level in the high and low FCGBP expression groups in the MESO cohort. (**J**–**L**) Immune cell infiltration level in the high and low FCGBP expression groups in the PAPG cohort. (**M**–**O**) Immune cell infiltration level in the high and low FCGBP expression groups in the SARC cohort.
